# Supplementary material for: The interactome of intact mitochondria by cross-linking mass spectrometry provides evidence for coexisting respiratory supercomplexes
Source: Mol Cell Proteomics. 2017 Dec 8;17(2):216–32. doi: 10.1074/mcp.RA117.000470 (PMC5795388; doi:10.1074/mcp.RA117.000470)
Supplement: Supplemental Data [file supp_RA117.000470_133922_0_supp_23978_fzffwf.zip › spectra_annotation/mito_spectra_annotation/209-1-22-1-11-1.pdf]

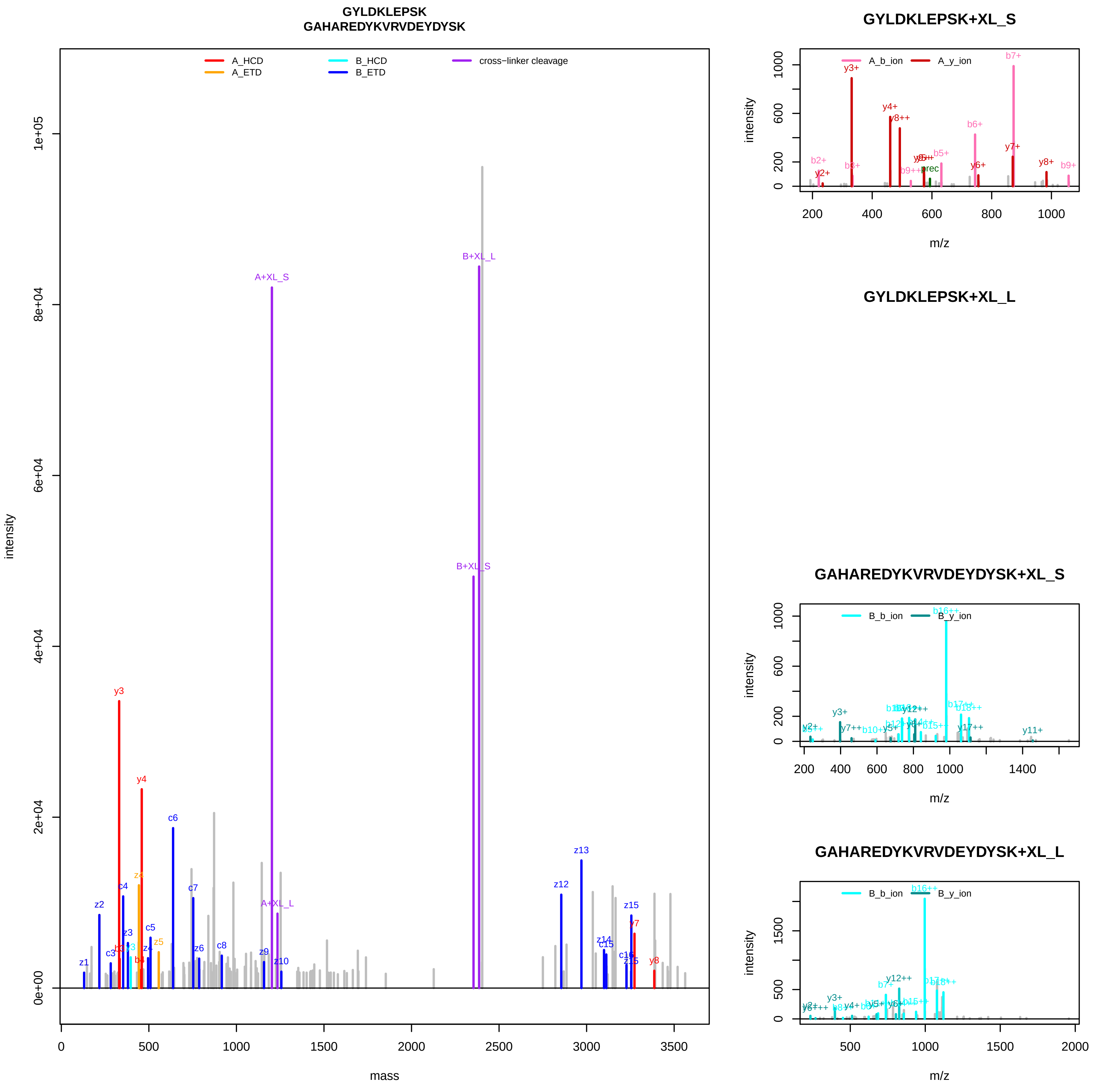

GYLDKLEPSK
GAHAREDYKVRVDEYDYSK
A_HCD
A_ETD
B_HCD
B_ETD
cross−linker cleavage
intensity
mass
0e+00
2e+04
4e+04
6e+04
8e+04
1e+05
0
500
1000
1500
2000
2500
3000
3500
A+XL_S
B+XL_L
B+XL_S
A+XL_L
y3
y4
c6
z13
z12
z15
y7
y8
z1
z2
c3
c4
z3
c5
z5
c7
z6
c8
z9
z10
z14
c15
GYLDKLEPSK+XL_S
A_b_ion
A_y_ion
b7+
y3+
y4+
y8++
b6+
y7+
b5+
b2+
y2+
b3+
y6+
y8+
b9+
b9++
prec
m/z
200
400
600
800
1000
GYLDKLEPSK+XL_L
GAHAREDYKVRVDEYDYSK+XL_S
B_b_ion
B_y_ion
b16++
y3+
y2+
y7++
b10++
y5+
y12++
b17++
b18++
b15++
y17++
y11+
1400
GAHAREDYKVRVDEYDYSK+XL_L
1500
2000
y4+
b7+
